# The effect of total knee arthroplasty on patients’ balance and incidence of falls: a systematic review

**DOI:** 10.1007/s00167-016-4355-z

**Published:** 2016-10-19

**Authors:** M. Moutzouri, N. Gleeson, E. Billis, E. Tsepis, I. Panoutsopoulou, J. Gliatis

**Affiliations:** 1 Department of Physiotherapy, Branch Department of Aigion, Technological Educational Institute (TEI) of Western Greece, Psaron 6, 25100 Aigion, Greece; 2 School of Health Sciences, Queen Margaret University, Musselburgh, UK; 3 Orthopedic Surgery Department, University Hospital of Patras, Rio, Greece; 4 Queen Margaret University, Musselburgh, UK

**Keywords:** Balance control, Falls, Falls risk, Total knee arthroplasty, Systematic review

## Abstract

**Purpose:**

Despite the high incidence of falls in patients with OA, few studies have explored whether falls risk is affected after patients undergo total knee arthroplasty (TKA). Therefore, the aim of this systematic review was to identify the extent of the effects of TKA on balance and incidence of falls by critically reviewing the available literature.

**Methods:**

A systematic review of published literature sources was conducted up to March 2014. All studies assessing balance and incidence of falls after TKA (without physiotherapeutic intervention) were included. The methodological quality of each study was reviewed using the Critical Appraisal Skill Programme tool.

**Results:**

Thirteen studies were included, comprising of ten cohort studies (Level II) and three studies with Level of evidence III.

**Conclusions:**

Findings provide evidence that TKA improves significantly single-limb standing balance (~60%) and dynamic balance up to 1-year following surgery (Level of evidence II). Moreover, TKA influences positively fear of falling and incidence of falls by switching 54.2 % of pre-operative fallers to post-operative non-fallers (Level of evidence II–III). It is highlighted that knee extension strength, proprioception and symmetrization of postural strategies have not fully recovered post-TKA and influence balance performance. Clinically, these persistent deficits need to be mitigated by physiotherapy even before TKA takes place.

## Introduction

Balance is essential for maintaining postural stability while performing functional activities and for fall avoidance [48]. Balance (dynamic and static) is a complex function which requires integration of sensory information regarding the position of the body and the ability to make appropriate motor response to body movement [22]. More precisely, it depends on sensory inputs from somato-sensory (proprioception), visual and vestibular systems [5], as well as, response of muscles. Static balance refers to maintaining equilibrium while standing in one spot, whereas dynamic balance involves motion and is defined as maintaining equilibrium during locomotion [37]. Falls and loss of balance most commonly occur during movement-related tasks such as walking and less frequently during static activities [23].

Balance deficits have been identified as one of the integral components impairing daily living in patients with knee osteoarthritis (OA) and are associated with an increased risk of falls and poor mobility [59]. Approximately 60–80 % of patients with knee OA report knee instability, which causes activity limitations [14, 44]. Osteoarthritis has been shown to be an important risk factor for falls with more than 40 % of all patients and 64 % of female patients, with OA reporting falls within a year in America [18, 59]. Potential mechanisms causing balance impairments in this population have not yet been fully elucidated [13, 25, 39, 50, 51]. Age-related impairments in the capacities of physiological systems controlling balance is one of the potential contributory mechanisms [47]. Proprioceptive impairment of the joint sometimes precedes knee OA and deteriorates further the degeneration associated with the disease [46]. Knee pain and quadriceps’ weakness are associated with increased postural sway [8, 20, 27, 30]. However, while total knee arthroplasty (TKA) (treatment of choice for end-stage OA) aims to relieve pain, correct deformities and restore loco-motor function, it is not established whether it has an effect on patients’ balance and incidence of falls. The literature suggests that patients with knee OA undergoing TKA will often present with a substantial loss of balance control and proprioceptive acuity that is frequently precipitated by a lack of confidence [13, 25, 28, 31, 34, 35, 39, 42, 51]. Despite the high incidence of falls in this population, there is a scarcity of investigations in the literature focusing on the risk of falls and subsequent impairments in function for patients with knee OA after undergoing TKA.

Chronic knee OA pain is reduced after TKA, but little is known about the recovery of proprioceptors, neuromuscular control, joint-related stability and also about each aspect’s natural recovery after surgery. Conversely, asymmetrical gait patterns and postural sway (in the coronal plane) combined with increased forward trunk movement (in the sagittal plane), observed especially in the early post-operative period, cause balance difficulties and increased risk of falls [10, 19, 24].

Residual physical deficits have been observed up to 7-year following TKA, with significant impact on functional status (i.e. postural stability, walking speed, stair ascent/descent) [6, 17, 36, 41, 55, 56, 60, 61]. In turn, decreased muscle strength, ROM and altered movement patterns evident post-surgery affect the sensory and mechanical function of the joint. Byrne & Prentice [7] reported that TKA affects the ability of patients to step over an obstacle.

Thus, there are a number of factors that may influence the effect of TKA on balance and consequently the incidence of falls. Understanding of the mechanisms associated with the recovery of the systems that control balance and the specific residual problems after surgery may ultimately help to enhance the design of rehabilitation programmes using approaches that are justified by scientific evidence. Based on this rationale, the novel aim of this study was to conduct a systematic review in order to identify the effects of TKA on balance and on the incidence/ risk of falls.

## Materials and methods

The electronic databases: the Cochrane Central Register of Controlled Trials (Cochrane Library), MEDLINE, EMBASE (via ProQuest), Biomed Central, CINAHL (via EBSCO host) and Physiotherapy Evidence Database (PEDro) were searched from January 1995 to the present (September 2014). The MEDLINE Mesh keywords used were: Balance OR stability OR postural control OR falls AND knee replacement OR knee arthroplasty in the title or abstract or keywords of the studies. Clinical trials published in the English language were included. The reference lists of all eligible papers were also screened to identify any studies that had been missing from the databases. The format of the search terms was modified appropriately for use in each database searched.

Eligibility assessment was performed independently in a standardized manner, and disagreements amongst reviewers were resolved by consensus. Therefore, studies were included if they fulfilled the following 4 criteria:

1. Participants underwent primary TKA.
2. No physiotherapeutic intervention/rehabilitation was involved after hospital discharge for TKA.
3. Balance, postural control and/or falls incidence was/were used as outcome measure/s.
4. The full paper was published in the English language.

Studies included cross-sectional, cohort and randomized controlled trials (RCTs), but excluded case studies. All cadaver or animal studies were excluded. Moreover, studies with samples involving patients with rheumatoid arthritis (RA) were excluded.

Two evaluators independently selected the studies based on titles and abstracts and excluded those not related to the subject. The full text was obtained for all papers that were considered potentially relevant. Once collected, these were reviewed by both reviewers to determine whether eligibility criteria had been fulfilled. The studies finally included were analysed according to a certain structure: author/year, sample, study design, assessment outcome measures, timeline, physiotherapy treatment, equipment and effects. The selection criteria were applied to the title and to the abstract of all articles retrieved in the search of the literature. The full text articles not excluded in the initial selection process were then evaluated for inclusion using the same eligibility criteria.

The methodological quality of each study was evaluated according to the Critical Appraisal Skills Programme (CASP) tool. This appraisal tool has been widely used in systematic reviews and is recognized to be a valid tool. The tool uses a set of 11 questions to evaluate domains such as: study design, appropriateness of design, randomization method, blinding, accuracy in the description of the sample recruitment, treatment effects, finding’ interpretation.

In the first stage, a descriptive review of studies assessing balance and falls incidence in patients after knee replacement was undertaken (Table 1).

**Table 1:** Description of the included studies in the systematic review investigating balance and risk of falls in TKR patients

	Sample	Outcome measures	Timeline	Study findings	Clinically relevant findings to balance and falls
Cho and Hwang [12]	*N* = 11 (11F) No CG Age: 61.7±7.3 Inclusion: Radiographic varus deformity& medial compartment OA degeneration undergoing TKR	VAS; WOMAC; varus angle; SLSB in horizontal plane force platform maximum isometric peak torque of quadriceps	Pre-TKR and 11-day post-TKR	Improvements (~60%) in SLSB in patients with varus OA knees 11-day post-TKR	Improvement in SLSB post-TKR
				Poor pre-TKR SLSB associated with better SLSB post-TKR.	
Gage et al. [15]	EG: *n*= 8(2 M,6F) Age: 62.9±6.0 CG: n=9(5 M,4F) Age: 62.1±5.6 Inclusion: post-TKR patients, first right TKR, at least 6 months after surgery	EMG and kinematic responses with rotational sagittal plane perturbation platform	At least 6 months post-TKR	Dynamic balance not impaired in EG vs CG in sagittal plane	No difference between groups in dynamic balance in sagittal plane
				Whole body COM displacement not different between groups vs joint angle displacement and EMG => different strategy to maintain balance from CG.	
				EMG and kinematic responses in EG are bilateral despite unilateral joint disease	
Gage et al. [16]	EG: *n* = 8(6F) Age: 62.9±6.0. CG: n=9(4F) Age: 62.1±5.6 Inclusion: post-TKR patients, first right TKR, at least 6 months after surgery	EMG and kinematic responses with rotational frontal plane perturbation platform	At least 6-month post-TKR	Dynamic balance control impaired in EG vs CG in frontal plane	Impaired dynamic balance of EG vs CG in frontal plane
				Increased COM displacement in EG vs CG	
				Differences in joint angle displacement and EMG of EG vs CG => different strategy to maintain balance from CG.	
				EMG and kinematic responses amongst patients are bilateral despite unilateral joint disease	
Levinger et al. [29]	EG: *n* = 35 (16F) Age: 67±7. CG: n=27 (14F) Age: 65±11 Inclusion: patients with knee OA who could walk independently for 45 m undergoing TKR	QoL; WOMAC; Incidental and Planned Activity Questionnaire (IPAQ), Falls Efficacy Scale (FES-I), Physiological Profile Assessment (PPA) for falls risk	Pre-TKR & 4-month post-TKR	No significant difference in falls risk between groups post-TKR	Increased risk of falls in EG compared to CG.
				No significant difference in postural sway between groups	Impaired SLSB of EG vs CG
				QoL: significant reduced post-surgery.	
				Significant improvement in WOMAC post-TKR	
				Less strength and poorer proprioception for the EG post-TKR compared with the CG	
Mandeville et al. [32]	EG: n= 19(14F) Age: 64.0±7.74. CG: n=21(13F) Age: 63.1±4.26 Inclusion: end-stage OA undergoing TKR	VAS; WOMAC; obstacle overcoming; kinematic displacement on force platform during gait	Within 2 pre-TKR weeks and 6-month post-TKR	Improvement in WOMAC post-surgery	Impaired dynamic balance in EG vs controls
				Poorer gait stability in EG (smaller displacement COM/COG) than CG	
				EG and CG cross obstacles similarly	
Mauer et al. [33]	EG: n=29(19F) Age 72, 6±5,4, bilateral (BL) TKR. CG: n=27(17F) Age 70, 6±5,5. Inclusion: knee OA who could climb stairs, rise form a chair, have 20/40 vision or better undergoing TKR	Balance (SLSB for 30 s); Obstacle avoidance success rate	EG tested post-TKR: 2,75±1,29 (range: 1–5 years)	EG SLSB duration was 67% less than the CG	Impaired SLSB in EG vs CG.
				EG 30% less obstacle avoidance success rate than the CG	Increased risk of falls in EG vs CG
McChesney and Woolacot [11]	N=22 Age:≥70 Groups: knee OA, ankle OA, patients undergoing TKR	TJPS; EMG and kinematic responses with force platform	Not stated	Ankle & knee groups with lower TJPS showed increased COP variance.	No difference on SLSB or dynamic between groups
				Post-TKR patients showed no reductions in any aspect of postural control.	
Quagliarella et al. [43]	N=240(142F). EG1: n=81 THR Age range: 40–80/42–82 years EG2: n=100 TKR Age range: 48–80/48–79 years CG: N=59 Age 67.4±5,9. Patients able to stand without support for 120 s.	Posturography on force plate	Pre-op; 6 months &12 months post-TKR	No statistically significant improvement in posturographic parameters in EG1 & EG2 vs CG group at follow-ups post-TKR	Standing balance did not show a clear trend towards improvement in TKR patients post-TKR
				Statistically significant improvement in pain and function of EG1 & EG2 post-TKR	
				Posturography not recommended as a method to evaluate balance in TKR patients	
Schwartz et al. [45]	n=62(52F) mAge: 73 (r: 57–83). Inclusion: Knee OA patients able to walk & follow simple instructions undergoing TKR	Dynamic & static Balance with force platform; TUG; SF-36	Pre-TKR & 12-month post-TKR	improved knee function& QoL	Significant improvement in dynamic and functional balance; NS for static balance
				improved dynamic balance	
				Improvements in static balance measures did not reach statistical significance.	
				Improved weight-bearing during squat	
Swinkels et al. [54]	n= 99(63F) Age 73.4±4.9 Inclusion: primary TKR	falls number; WOMAC; ABC-UK; GDS	Pre-TKR and 12-month post-TKR	~45% patients fall again in the first year post-TKR	Significant switch of pre-TKR fallers becoming non-fallers post-TKR
				Improved balance confidence, WOMAC and GDS post-TKR	
Swinkels and Allain [53]	n=22 (16F/ Age: 74,8 ± 5,2y) Inclusion: primary TKR.	falls number; WOMAC, ABC, GDS; BBS; TUG	2–50 day pre-TKR (mean: 23) 143–218 day post-TKR (mean: 183)	41% of patients exceeded MDC for BBS post-surgery	Functional balance improved post-TKR in 54% of patients
				50 % of patients exceeded MDC for TUG post-surgery	
				Findings on fallers are restricted by the small sample size	
Viton et al. [57]	20 patients EG1: N=8(3F) Age: 67 (46–77) CG N=12(6F) Inclusion: Unilateral TKR	VAS; kinetics/kinematics in side step on force platform	15-day pre -and 12-month post-TKR	Improved VAS	SLSB improved in operated limb during tasks
				Presented with more symmetrical posturomotor strategies	
				Evidence of persisting posturomotor impairments of EG vs CG	
				Increased SLSB in operated limb in EG	
Yakhdani et al. [60]	EG1: *n* = 14(10F) Age 61.6±10. CG: n=12(7F) Age 62.0±12.6 Inclusion: Unilateral knee ΟΑ undergoing TKR, sufficient physical condition	Dynamic balance; falls; gait variability	pre-TKR, at 6 weeks, at 6- and 12-month post-TKR	EG1: increased maximum gait speed post-TKR	Reduced risk of falls.
				Decreased gait variability of EG post-TKR in relevance to CG	Recovery of dynamic balance
				Increased stability post-TKR	

*CG* control group, *EG* experimental group, *TKR* total knee replacement, *mCTSIB* modified clinical test for sensory interaction and balance, *TJPS* threshold of passive joint position sense,* ABC-UK* activity balance confidence scale, *GDS* geriatric depression scale, *BBS* berg balance scale, *MDC* minimal detectable change, *COP* centre of pressure, *COG* centre of gravity, *TUG* timed up and go test, *NS* non significant

In the next stage, the analysis involved a critical appraisal process of the studies according to the CASP tool to determine the methodological quality and to summarize findings (Table 2). Strong evidence was indicated by the availability of consistent findings in two or more high quality RCTs, moderate evidence by a high quality RCT, or two or more low quality RCTs. Limited evidence was indicated by cohort studies and case–control studies.

**Table 2:** CASP Checklist for the studies included in the current systematic review

	Cho et al. [12]	Gage et al. [15]	Gage et al. [16]	Levinger et al. [29]	Mandeville et al. [32]	Mauer et al. [33]	McChesney and Woolacot [11]	Quagliarella et al. [43]	Schwartz et al. [45]	Swinkels et al. [54]	Swinkels et al. [53]	Viton et al. [57]	Yakdhani et al. [60]
Clearly focused issue	✓	✓	✓	✓	✓	✓	✓	✓	✓	✓	✓	✓	✓
Acceptable patient recruitment	✓	✓	✓	✓	✓	✓	✓	✓	✓	✓	✓	✓	✓
Randomized patient assignment	NA	✓	NA	✓	✓	NA		✓	NA	✓	✓	NA	NA
Sample based on power calculation	✓	✗	✗	✗	✗	✗	✗	✗	✗	✓	✓	✗	✗
Patients fulfilled follow-ups	✗	✓	NA	✓	✓	NA	✓	✓	✗	✗	✗	✓	✗
Patients, examiners blinded	✓	✗	✗	✗	✓	NS	NS	✗	NA	✓	✓	NS	NS
Similarity of patients/groups	✓	✓	✓	✓	✓	✓	✓	✗	NA	✓	✓	✓	✓
Equal treatment of patients/groups	✓	✓	✓	✓	✓	✓	✓	✓	✓	✓	✓	✓	✓
Clearly specified outcome measures	✓	✓	✓	✓	✓	✓	✓	✓	✓	✓	✓	✓	✓
Large treatment effect	✓	✓	✓	✓	✓	✓	✓	✗	✓	✓	✗	✗	✓
Estimate treatment effect (CI)	✓	NS	NS	✓	✗	✓	✗	✓	✗	✗	NS	NS	NS
Appropriate results analysis	✓	✓	✓	✓	✓	✓	✓	✓	✓	✓	✓	✓	✓
Confounding factors listed	✓	✗	✗	✓	✓	✓	✗	✗	✓	✓	✓	✗	✓
Appropriate interpretation of results	✓	✓	✓	✓	✓	✓	✓	✓	✓	✓	✓	✓	✓
Generalization of results	✓	✗	✗	✗	✗	✗	✗	✗	✗	✓	✗	✗	✗
Applicable to clinical practice	✓	✓	✗	✓	✓	✓	✓	✓	✗	✓	✓	✗	✓
Results coincide with relevant literature	✓	✓	✓	✓	✓	✓	✓	✗	✗	✓	✓	✗	✓
Our evaluation	Reasonable. Short-term follow-up	Reasonable. Potential bias due to small sample size	Reasonable. Potential bias due to small sample size.	Good	Good	Good	Reasonable. CI not reported.	Outcome measures used not recommendable for TKA.	Reasonable. Potential bias due to large losses to follow-up	Reasonable. Considerable losses to follow-up	Reasonable. Potential bias due to small sample size	Reasonable. Potential bias due to small sample size	Reasonable. Potential bias due to small sample size

All data extracted from the studies were analysed independently by each reviewer (MM and RP) and subsequently discussed. In any case of disagreement, further discussion was performed with a third reviewer (NG) to reach a mutual agreement.

### Statistical analysis

Studies comparing static and dynamic balance pre- and post-TKA with comparable outcome measures were identified and pooled through a meta-analysis. Heterogeneity was assessed using the I^2^ measure. It was considered that I^2^<60 % was acceptable to pool data [21]. The statistical significance was considered at *p* < 0.05. Qualitative review of the evidence was performed when the studies could not be pooled.

## Results

### Search results

**Fig. 1:**
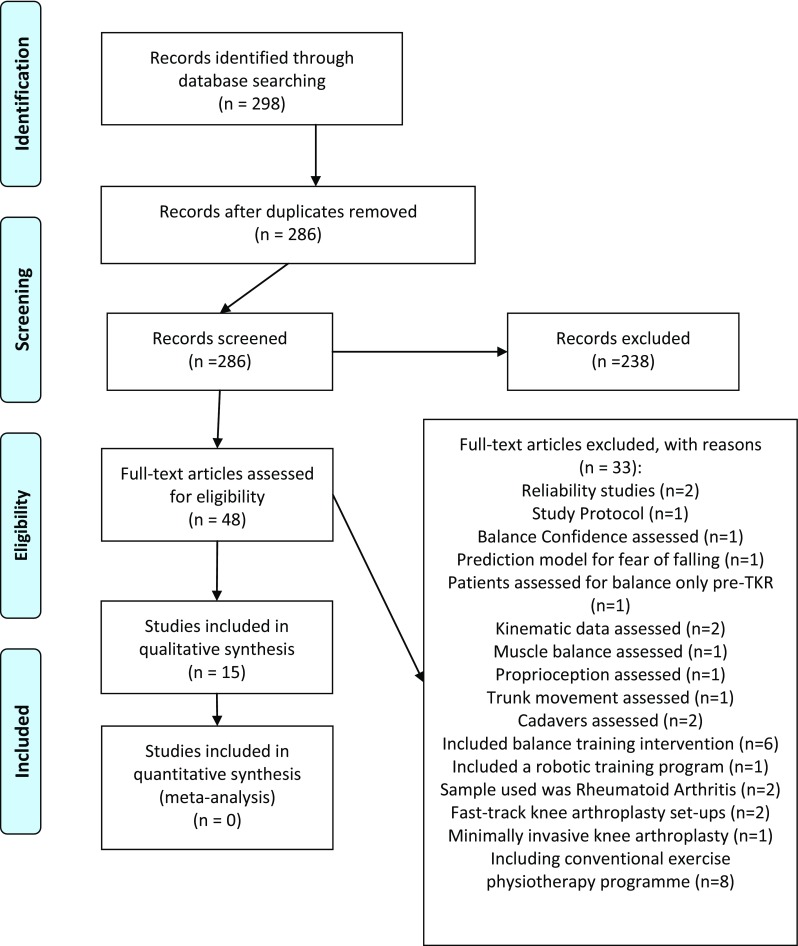
PRISMA Flow Diagram to depict search strategy results. From: Moher D, Liberati A, Tetziaff J, Altman DG, The PRISMA Group (2009) Preferred reporting items for systematic reviews and meta-analysis: the PRISMA statement PLoS Med 6(6): e1000097. doi:10.137/journal.pmed1000097

A total of 276 citations were identified by the search strategy, summarized in Fig. 1. Initially, 237 studies were eliminated because the title, abstract or keywords did not match the proposed theme. Of the 36 that remained, 22 were eliminated due to the non-English language used. Therefore, due to the other aforementioned exclusion criteria, 13 studies were deemed eligible and were finally included in the review.

### Cohort characteristics

In all studies, patients had primary OA (grade III–IV according to the Kellgren and Lawrence system) and fulfilled criteria to undergo TKA. All knees were implanted with the same type of cemented prosthesis (unilaterally or bilaterally). No patellar component was inserted in any of the studies.

A description of the included studies, with the outcome measures used, the follow-up period and the clinically relevant findings is presented in Table 1.

### Outcome measures

All studies used validated measures to assess balance parameters [48]. Functional stability limits, reactive control, control of balance during an active task, standing balance are all balance components being investigated in the studies, all linked with balance-related falls [29].

Regarding the incidence of falls after TKA, five studies included risk of falls assessment in addition to balance assessment [16, 29, 53, 54, 60]. One study used the short form of the Physical Profile Assessment (PPA) that encompasses five tests (proprioception, knee strength, postural sway in two directions and reaction time) to assess risk of falls [29].

### Critical appraisal of studies’ methodological quality

Of the 13 studies included in the systematic review, 10 followed a cohort design (Level IIc), seven of which included a control group [11, 12, 29, 32, 43, 57, 60]. Three studies were observation case–control studies (Level III) [15, 16, 54]. The quality of the studies has been assessed as although all studies satisfied a similar number of criteria, their methodologies varied substantially.

All studies offered clearly defined research questions, population’ characteristics and methods of assessing balance (Table 2). In 5 studies [12, 45, 53, 54, 60], a number of participants were lost to follow-ups, implying potential bias. Only 3 studies had based their sample on a power calculation analysis [12, 53, 54]. In relation to interpretation, all studies discussed their findings based on current evidence. Generalization of findings was feasible in only 2 studies [12, 54], as in the other ones, either the sample size was not sufficient, or control group was absent.

### Synthesis of results

#### Static balance post-TKA

Patients with TKA presented with 67 % less (*p* < 0.05) mean single-leg stance duration than that of healthy controls [33]. Postural sway in static single-limb stance was improved ~60 % 11 days after TKA compared to pre-surgery [12]. When balance was perturbed in a sagittal plane, no difference in balance control was observed between TKA patients and age-matched controls [15]. However, when balance was perturbed in the frontal plane, control of balance showed statistically significant impairment in TKA patients compared to controls [16].

#### Dynamic balance post-TKA

During a dynamic task, patients with bilateral TKA present with a mean obstacle avoidance success rate that was 32 % less than that of the control group [33]. During tasks such as stepping down, lateral steps, obstacle crossing, the success rate was increased after TKA. However, statistically significant conservative strategies (slower speed, shorter stride length, shorter base of support) (*p* < 0.05) were adopted resulting in increased duration of each task of up to 30 % [12, 32, 33, 45, 57].

#### Falls risk

After TKA, less than half (45.8 %) of pre-operative fallers continued to fall [54]. Patients who fell pre-operatively had an eightfold increase in the risk of post-operative falling [54]. A lower risk of falls was reported in 4 studies after surgery [29, 53, 54, 60]. In the PPA risk of falls, the only parameters that reached statistical significance were proprioception and knee extension strength 1-year post-surgery [29]. Balance confidence (ABC-UK) was significantly improved after TKA; however, results remained statistically significant (*p* < 0.001) only in patients with no history of falls pre-operatively [54]. Patients with higher ABC-UK pre-operatively reduced the odds of becoming a faller for up to 1 year post-operatively by 98 % (95 % CI 0.96–1.01, *p* = 0.04) [54]. Berg Balance Scale scores of fallers and non-fallers were similar both before and after TKA, although scores were improved more than the minimal detectable change (MDC) in 41 % of TKA patients [53].

## Discussion

The most important finding of the present study was that TKA influences positively (a) fear of falling and incidence of falls by switching 54.2 % of pre-operative fallers to post-operative non-fallers and (b) balance for up to 1-year following surgery. The rationale for the study was that by analysing the available literature, an understanding might be promoted of how mechanisms controlling balance, compensate or respond after surgery, and in which timeline this occurs.

Thirteen studies fulfilled criteria that had been set and were ultimately used in the analysis. No study involving post-hospital discharge physiotherapy intervention or, any other type of rehabilitation training, which might otherwise confound the extent of the isolated influence of surgery was included in this review. Despite a large number of studies in this field, very few offered a high level of evidence (Level of evidence < IIc). The sample in the studies comprised of 652 individuals in total (167 being controls), with a mean age of 71.4 years; recruited patient’ characteristics were typical of middle-aged individuals undergoing TKA for knee OA and were therefore considered to be representative of TKA population. The methodological quality of the studies, as assessed by the CASP scale, was acceptable. However, due to variability in sampling procedure and the absence of power calculation analysis in most studies, external validity and therefore generalizability have been limited. Major drawbacks in the studies were the lack of randomization and the lack of a control group in some studies.

Regarding the balance effects found in most studies, there was a significant balance improvement (p < 0.001) in both tasks and confidence after TKA compared to the pre-operative state. While balance and sensori-motor performance were not fully restored after TKA, postural responses began to normalize in both quiet stance and dynamic tasks [32, 33, 57]. Static balance did not show a clear trend towards improvement [43]. Single-leg standing balance improved up to 60 % post-TKA, but remained poorer than age-matched controls for up to 1 year [12, 29, 33, 45, 57]. Dynamic and functional balance was found to be improved 6-month post-surgery but again not fully recovered compared to age-matched controls [32, 45, 53, 54, 60].

In studies investigating balance and postural control, the clinically relevant outcome would be patients’ reported falls. A 24.2 % post-operative falls rate for TKA patients was reported up to 1 year, which is less than current estimates (33 %) for community dwelling older people [53, 54]. The rate although reduced remained as high as 45.8 % for individuals identified as fallers prior to surgery. Nevertheless, there was a significant switch of fallers’ pre-TKA who became non-fallers after TKA (54.2 %) [54]. At least one fall in the first year post-TKA was recorded for 48 % of the surgical group compared with 30 % of the control group [29]. Following TKA, there was a 27 % reduction in the number of patients exceeding the cut-off point of 14 s in Timed Up and Go (TUG) [53, 54]. This time cut-off point has been proposed as a criterion for ruling out a high risk of falls in older adults [47]. Therefore, although the likelihood of falls seems to decrease after TKA, there is still a considerable amount of falls recorded post-TKA.

During TKA, the replaced knee is deprived from a variety of key proprioceptors, which have been resected (ACL, cartilage, menisci, etc.). Moreover, oscillations used by the knee joint to regulate postural control are unlikely to reach a detectable threshold by sensory receptors in the replaced knee [9]. Presumably, extra-capsular proprioceptors need to compensate for the loss, and in order to maintain stability and balance, albeit at reduced levels of capability [1, 58]. Different types of prostheses and retention of the PCL also have an impact in joint translation and mobility components [2, 4, 49]. However, different type of surgery techniques (posterior stabilization versus posterior cruciate ligament-retained) has shown contradictory findings on whether they influence balance and proprioception [3, 5, 52]. The addition of a patellar (prosthetic) component may further influence afferent sensory input; however, no relevant study was identified in the literature. Skinner *et al.* [49] suggested that the loss of proprioception due to arthritis was not improved by surgery. By contrast, Barrett et al. [4] claimed that when joint alignment and the ‘joint space height’ are reconstituted, the sense of position is improved, indicating that the reloading of lax collateral tissues at the time of the operation may be beneficial. Moreover, it has been shown that soft tissue balance (length-tension relationships for PCL and collateral ligaments) after surgery in both flexion and extension is important for allowing satisfactory post-operative knee proprioception [4]. Any difference in the tension of the medial and lateral collateral structures may therefore be perceived as a varus or valgus movement of the leg and may produce an antagonistic and corrective action from the hamstrings and quadriceps muscle groups, thus affecting proprioception [4]. Taking into account all the above literature, a number of factors could intrude as a result of surgery that could actually have an impact on patients’ neuro-sensory performance.

Bilateral postural responses after perturbation differ between TKA patients and age-matched controls. These differences are mostly observed in activation latency of muscles acting on the knee and in subsequent knee joint kinematics (reduced knee extension), suggesting a central postural re-organization process to protect against overly stressing the joint [15, 16, 32, 57, 60]. During walking, variability in knee kinematics is reduced and local dynamic stability again seems to be gradually restored [60]. Therefore, the mild improvements observed in balance following TKA may result from the retensioned capsuloligamentous structures and reduced pain and inflammation [52]. Patients post-operatively tend to normalize their weight distribution and develop more symmetrical posturomotor control.

### Implications for clinical practice

Clinically, from a surgery perspective, correcting knee joint alignment and specifically varus deformity post-TKA has been shown to improve balance [12]. Considering the catastrophic consequences of peri-prosthetic fractures after a patients’ fall, 3D evaluations of the alignment and computer-assisted gap-balancing techniques, than conventional techniques of TKA, may produce more advantageous balance effect [26, 40]. The clinical relevance regarding rehabilitation is that patients’ training should involve rehabilitative strategies in static and dynamic tasks to achieve symmetrical weight distribution, implemented both pre- and post-TKA. Interestingly movement and weight distribution symmetry training, via the use of biofeedback, was recently introduced in the literature [62]. At the moment, there is only preliminary evidence to underpin the use of targeted sensori-motor elements within a physiotherapy programme [38]. Bilaterally observed impairments indicate that rehabilitation should include balance exercises involving both single- and double-leg stances to provoke overload and adaptation and prevent falls. Balance perturbation tasks can be more targeted towards frontal plane provocation and less in the sagittal plane [15, 16]. Moreover, knee strength and proprioception showed statistically significant improvement after TKA, whereas postural sway with eyes open and reaction time did not [29]. Finally, physiotherapy programmes should mostly incorporate the influential factors of falls (i.e. knee strength, proprioception and balance exercises with eyes closed).

Several limitations need to be considered when interpreting the findings of this review. Because of the methodological heterogeneity across the study designs (i.e. in methodological outcome measures and timing of measurements), results from a potential meta-analysis (with an *I* ^2^>85 %) would not have added consistency of evidence. Furthermore, due to methodological flaws across the studies, the level of evidence was not high enough to allow the generalization of results. More studies with robust methodologies are needed to investigate the effect of TKA on balance and falls incidence.

## Conclusions

The findings of this systematic review provide moderate evidence to support that TKA influences balance positively for up to 1-year following surgery. Studies offering Level of evidence II showed up to 60 % improvement in standing balance as early as 11-day post-TKA. Moreover, TKA influences positively fear of falling and incidence of falls by switching 54.2 % of pre-operative fallers to post-operative non-fallers (Level of evidence II–III). Patterns of change (acute and chronic) and congruence amongst the interpretation of findings from the reviewed papers endorse a conceptual framework for the knee undergoing TKA surgery. The framework supports that knee extension strength, proprioception deficits and compensatory postural strategies are persisting after surgery and are acting as the potential factors contributing to why balance and falls might be linked and only partially restored after TKA.
